# Percutaneous Transluminal Angioplasty with Stent Placement versus Best Medical Therapy Alone in Symptomatic Intracranial Arterial Stenosis: A Best Evidence Review

**DOI:** 10.7759/cureus.2988

**Published:** 2018-07-16

**Authors:** Arjun Padalia, Jacob A Sambursky, Colby Skinner, Mouwafak Moureiden

**Affiliations:** 1 College of Medicine, University of Central Florida, Orlando, USA; 2 University of Central Florida College of Medicine, Orlando, USA

**Keywords:** ptas, icas, stent, bare metal, drug eluting, self expanding, stroke, intracranial stenosis

## Abstract

Intracranial arterial stenosis (ICAS) is a common cause of stroke, and the risk of ischemic stroke from a stenotic intracranial artery remains high despite best medical therapy (BMT). As a result, clinicians have increasingly turned to percutaneous transluminal angioplasty and stenting (PTAS) over the last decade as an alternative therapy in high-risk patients with symptomatic ICAS. In this review, we will critically analyze multiple clinical trials to assess the safety and efficacy of PTAS with BMT versus BMT alone. The Stenting and Aggressive Medical Management for Preventing Recurrent Stroke in Intracranial Stenosis (SAMMPRIS) trial reported a higher rate of stroke or death within 30 days in the PTAS plus BMT group (14.7%) than the BMT only group (5.8%, p = 0.002). The rate of any stroke during the follow-up period (mean = 32 months) was higher in the PTAS plus BMT group (22.3%) than the BMT only group (14.1%, p = 0.03). The Vitesse Intracranial Stent Study for Ischemic Stroke Therapy (VISSIT) trial reported a higher rate of stroke or death within 30 days in the PTAS plus BMT cohort (24.1%) than the BMT only cohort (9.4%, p = 0.05). There was also a higher rate of hard transient ischemic attack (TIA) or stroke within one year in the PTAS plus BMT group (36.2%) than the BMT only group (15.1%, p = 0.02). The Vertebral Artery Ischaemia Stenting (VIST) trial reported the rate of any stroke during the follow-up period to be two events in 50 person-years in the PTAS plus BMT cohort versus four events in 45 person-years in the BMT only cohort, with a calculated hazard ratio of 0.47 (p = 0.39). Vertebral Artery Stenting Trial (VAST) reported a higher incidence of stroke, MI, or vascular death in the PTAS with BMT cohort (22%) than the BMT only cohort (0%). Tang et al. reported no significant difference in the incidence of vascular events at one year and three years between the PTAS plus BMT and BMT only cohorts. However, the distribution of vascular events was more concentrated in the first postoperative week in the PTAS plus BMT cohort (75% of all vascular events) versus the BMT only cohort (17%). Feng et al. reported a lower periprocedural complication rate (9.1%) with the Enterprise stent in comparison to the Wingspan and balloon-expandable stents used in the SAMMPRIS and VISSIT trials. We conclude that PTAS should not be employed as first-line treatment in patients with symptomatic ICAS. However, PTAS should be considered in symptomatic ICAS patients that are hemodynamically unstable or have repeatedly failed BMT, especially at sites with lower rates of perioperative complications than those reported here.

## Introduction and background

Intracranial arterial stenosis (ICAS) is a common cause of stroke worldwide causing up to 54% of ischemic strokes in certain demographics [1-6]. The risk of ischemic stroke from a symptomatic stenotic intracranial artery is high despite best medical therapy (BMT) [7]. According to the WASID (Warfarin–Aspirin Symptomatic Intracranial Disease) trial, the one-year risk of ischemic stroke in the territory of a ≥50% stenotic intracranial artery was approximately 11-12% while on high-dose aspirin or warfarin therapy [8], however the rate increased to 23% when the degree of stenosis was ≥70% [9]. Clinicians have increasingly turned to percutaneous transluminal angioplasty and stenting (PTAS) over the last decade as an alternative therapy in high-risk patients with symptomatic ICAS [10,11]. However, despite the technical success of PTAS in patients with symptomatic ICAS, it has been less efficacious than BMT in randomized controlled trials (RCT) [12-15]. This may be partly attributed to the widely varying rate of periprocedural complications (4.4-50%) such as ischemic or hemorrhagic stroke, especially if there have been prior infarcts in the area of intervention [16,17]. Unfortunately, current efforts to reduce periprocedural complications have been shown to be inadequate as solutions to procedural complications have not been adequately addressed [18]. The aim of this review is to evaluate the safety and efficacy of PTAS plus BMT versus BMT alone in patients with transient ischemic attack (TIA) or cerebrovascular accident (CVA) secondary to ICAS using data from RCTs and other studies. We will evaluate the comparative rates of ischemic stroke in the territory of the stenotic artery, any ischemic stroke, intracranial hemorrhage, any stroke or death at multiple timepoints from intervention, and difference in complications rates based on stent type [19-21].

## Review

Aggressive medical treatment with or without stenting in high-risk patients with intracranial artery stenosis (SAMMPRIS), Derdeyn CP, et al. [12]

The Stenting and Aggressive Medical Management for Preventing Recurrent Stroke in Intracranial Stenosis (SAMMPRIS) trial was a large, multicenter RCT of 451 patients in the United States that assessed the difference in risk of recurrent stroke or death in patients with ICAS that underwent PTAS using the Wingspan stent plus BMT compared to patients that were managed with BMT alone. SAMMPRIS investigators defined BMT as aggressive medical therapy, which included aspirin (ASA) 325 mg daily for the entire follow-up duration, clopidogrel 75 mg daily for 90 days, and algorithm-based risk factor management targeting systolic blood pressure (SBP) < 140 mmHg (130 mmHg in diabetics) and low-density lipoprotein (LDL) < 70 mg/dL. SAMMPRIS investigators hypothesized that enrolled ICAS patients who received PTAS in addition to BMT would have a reduced risk of recurrent stroke or death compared to patients that were only managed with BMT.

Of the 451 patients enrolled, 224 patients underwent PTAS and were managed with BMT while 227 patients were managed with BMT alone. Participants had an average stenosis of 80.5% with 46.5% of patients having a 70-79% ICAS, 42% of patients having 80-89% ICAS, and 11.6% of patients having 90-99% ICAS.

Management of enrolled patients who have had a recurrent stroke is performed by two neurologists and a blinded adjudication committee. Two neurologists independently examined participants that have experienced a recurrent stroke. The first neurologist was aware of the patient’s treatment assignment while the second neurologist was not informed of a patient’s treatment assignment. The assessments of both neurologists were judged for appropriateness by an independent panel of blinded neurologist and non-neurologist physicians that were not aware of patient treatment assignments, and a final management plan for the patient was agreed upon.

Contrary to their hypothesis, SAMMPRIS investigators found that the risk of stroke or death at 30 days was significantly higher in patients receiving PTAS plus BMT than patients receiving BMT alone (14.7% versus 5.8%; p = 0.002). The theory behind the elevated incidence of periprocedural stroke was described as a complication due to embolized plaque [17]. At 30 days follow-up, there was an increase in the number of recurrent strokes and deaths associated with PTAS plus BMT than with BMT alone (14.7% and 5.8%, p = 0.06, respectively). At both the one-year and three-year follow-up, the PTAS plus BMT group had significantly more strokes or deaths relative to BMT only group (19.7% and 12.6% [p = 0.04] at year one, respectively; 23.9% and 14.9% (p = 0.02) at year three, respectively).

Derdeyn et al. suggest that the exaggerated difference at 30 days follow-up between PTAS plus BMT compared to BMT alone is responsible for the significantly different rate of stroke and death at one-year and three-year follow-up.

PTAS plus BMT for high-grade ICAS (70-99% occlusion) results in a significantly increased rate of recurrent strokes and death compared to patients receiving BMT alone. However, in patients with less severe stenosis (<70%), previous studies have found that stenting plus BMT led to improved mortality outcomes [22-25]. SAMMPRIS authors attribute the discrepancy between their initial hypothesis and results to the higher degree of stenosis in the enrolled patients; SAMMPRIS only enrolled patients with 70-99% stenosis but previous studies demonstrating the effectiveness of PTAS plus BMT enrolled patients with stenosis between 50 and 99%.

Strengths of this study include the randomized design, large sample size, multicenter data collection, and high generalizability. A frequent criticism of the SAMMPRIS trial revolves around the use of single versus double antiplatelet therapy in the setting of ICAS. Trial investigators gave both ASA and clopidogrel to all enrolled patients for antiplatelet therapy as part of BMT. However, there have been no studies that show the therapeutic efficacy of single antiplatelet therapy compared to dual antiplatelet therapy in symptomatic ICAS patients. Further, the ASA dose used in this trial (325 mg) is generally the dose used in stroke prevention with atrial fibrillation or acute stroke management. The results of studies performed in multiple vascular indications suggest the optimal dose to be 75-100 mg/d for stroke prevention in the absence of atrial fibrillation.

Effect of a balloon-expandable intracranial stent vs medical therapy on risk of stroke in patients with symptomatic intracranial stenosis (VISSIT), Zaidat OO, et al. [13]

The Vitesse Intracranial Stent Study for Ischemic Stroke Therapy (VISSIT) trial was a large, multicenter RCT of 112 patients in the United States funded by Micrus Endovascular Corporation shortly after the SAMMPRIS trial had been initiated. The study aimed to compare the safety and efficacy of PTAS using the balloon-expandable stent plus BMT versus BMT alone in patients with symptomatic ICAS. BMT included 81-325 mg of ASA daily for the entire length of the study and 75 mg of clopidogrel daily for three months. In addition, the patients’ individual risk factors were addressed using antihypertensives, statins, smoking cessation, and diet modification.

Patients considered for the study were those 18 years of age or older with symptomatic stenosis (70-99%) of the major intracranial arteries (Internal carotid artery, middle cerebral artery, intracranial vertebral, or basilar) and had experienced a TIA or stroke attributable to the target lesion within the past 30 days. Patients were excluded if they had a potential cardiac embolism, modified Rankin Scale (mRS) > 3, unstable neurological status, or coexisting intracranial pathology. A total of 112 patients were enrolled and randomized 1:1 using a telephone interactive voice response system; randomization was stratified by age and enrollment site. The two groups shared similar baseline characteristics including medical comorbidities, smoking history, blood pressure, LDL levels, body mass index (BMI), etc. The PTAS plus BMT group and BMT only group were similar in mean age (both, 61.8 years) and degree of stenosis (78.9% versus 80.4%).

Of the 112 patients who were randomized, 59 were assigned to receive a stent and 53 were assigned to receive medical therapy. One patient was excluded from analysis after it was revealed the patient did indeed meet exclusion criteria. All other patients were included in intention to treat analysis of their respective assigned groups.

Patients underwent a neurological evaluation including National Institutes of Health Stroke Scale (NIHSS) and mRS on initial discharge/postprocedure. The evaluation was performed by an NIHSS-certified investigator not involved in the procedure. Repeat evaluations were performed during follow-ups. The trial was unable to be double-blinded due to the inability to mask the stent group; however, neurological evaluation by an independent NIHSS investigator who was not involved in the study procedure decreased potential bias. Unlike the SAMMPRIS trial, which appointed a clinical committee to ensure the full clinical and angiographic criteria were met, the VISSIT trial declined to appoint a committee.

Due to the published poor outcomes in the SAMMPRIS trial, the sponsor of the VISSIT trial decided to run a premature analysis of the collected data. Enrollment in the trial was discontinued due to the low probability of observing a favorable outcome with PTAS plus BMT versus BMT alone.

The risk of hard TIA, stroke, or death at 30 days was significantly higher in patients receiving PTAS plus BMT than patients receiving BMT alone (24.1% versus 9.4%; p = 0.05). At one year, the PTAS plus BMT group still had a significantly higher risk of hard TIA or stroke relative to BMT only group (36.2% versus 15.1%; p = 0.02). Also, more patients in the PTAS plus BMT group recorded a worsening of their disability score (mRS) compared to the BMT only group after one year (24.1% versus 11.3%, p = 0.09). The authors concluded that in patients with symptomatic ICAS, PTAS with a balloon-expandable stent in addition to BMT increased 30-day and 12-month risk of TIA, stroke, and death relative to BMT alone.

Strengths of this study include the randomized design, large sample size, multicenter data collection, and high generalizability. Potential weaknesses of the study include the lack of a blinded adjudication committee to analyze primary and secondary endpoints as well as lower power and shorter follow-up time due to early cessation of enrollment. Lastly, the inconsistent dosage of ASA, including but not limited to the increased dosage (325 mg), is also a weakness of this trial.

Stenting for symptomatic vertebral artery stenosis: the vertebral artery ischaemia stenting trial (VIST), Markus HS, et al. [14]

The Vertebral Artery Ischaemia Stenting Trial (VIST) was a large, multicenter RCT of 182 patients in the United Kingdom that investigated the level of risk associated with PTAS plus BMT versus BMT alone in patients with symptomatic vertebral artery stenosis. Those with symptoms of a posterior cerebral circulation TIA or non-disabling stroke and vertebral artery stenosis ≥ 50% were recruited for the study.

Originally, patients were mandated to have symptoms within the last six months, but this requirement changed mid-trial when other studies reported the greatest risk of stroke was within the first three months of symptom development. Patients were excluded from the trial if the ischemic attack was secondary to a vertebral artery dissection, if the location of the vertebral stenosis was inaccessible, or if the patient was pregnant or lactating.

Prior to randomization, evidence of a vertebral artery occlusion had to be visualized with magnetic resonance angiography (MRA), computed tomography angiography (CTA), or intra-arterial digital subtraction angiography (DSA) by two experienced neuroradiologists. If there was doubt of the presence of stenosis, the patient would undergo two imaging modalities and both had to result in evidence of stenosis. The stenosis percentage was calculated with the North American Symptomatic Carotid Endarterectomy Trial (NASCET) method or the WASID measurement (if the distal vessel was not accessible). Randomization was completed by an online system.

The VIST investigators estimated that 245 patients for both arms of the study (BMT versus PTAS with BMT) were necessary in order to achieve 95% confidence at a power of 80%, but randomization was ceased at 182 patients due to slow recruitment and loss of funding. Ninety-one patients underwent randomization into the PTAS plus BMT group, 88 patients were randomized into the BMT only group, and three withdrew consent. Among the 91 randomized into the PTAS plus BMT group, 30 were excluded; the most common reason for exclusion was stenosis < 50% on DSA. The randomization was also stratified by the location of the stenosis, as the methods of treatment and overall risks are different. Of the 61 patients approved for PTAS plus BMT, 13 were to undergo intracranial PTAS. Follow-up checkpoints were completed at one month, six months, one year, and two years. When an endpoint was achieved, they were removed from the study.

The medical management was monitored by a consultant neurologist/stroke physician. PTAS was completed by a consultant interventional radiologist with experience of at least 50 stenting procedures, 10 of which were cerebral vessels. If it was clinically indicated, patients received BMT. BMT included antiplatelet, anticoagulation therapy, statin therapy, antihypertensive therapy, or diabetes mellitus therapy. All patients who underwent PTAS received dual antiplatelet therapy (clopidogrel and ASA) during the procedure. Dual antiplatelet therapy was continued for one month after the procedure. After one month of dual antiplatelet therapy, standard antiplatelet therapy was initiated. Physicians and patients were not blinded to treatment, but the study utilized an independent adjudication committee to analyze the primary and secondary endpoints, as they were masked to treatment randomization.

The primary endpoint of a fatal or nonfatal stroke in any artery during the two-year follow-up period was analyzed for those who underwent PTAS plus BMT and for those who received BMT alone. The VIST investigators reported four events in 45 person-years in the BMT group and two events in 50 person-years in the PTAS with BMT group, with a calculated hazard ratio of 0.47 (p = 0.39). Contrary to the SAMMPRIS and VISSIT trials, there was no difference in the risk of stroke during the two-year follow-up period for either study arm. The VIST investigators also investigated the important secondary outcome of periprocedural stroke incidence in those who underwent intracranial stenting. The complication rate within the first 30 days was higher for those who underwent stenting in addition to BMT than those who received BMT alone. The conclusion of increased risk with PTAS was consistent with the data reported in the SAMMPRIS trial.

Strengths of this study include the randomized design, multicenter data collection, the utilization of a blinded adjudication committee to analyze primary and secondary endpoints, losing no patients to follow-up, and no difference in the intensity of medical treatment (statin and antihypertensive treatment) for risk factor reduction between the two study arms. Potential weaknesses of the study include the low number of patients recruited compared to the pre-protocol analysis, low power of the study due to early cessation of enrollment, the lower number of intracranial stent placements relative to extracranial stent placement, and the increased use of dual antiplatelet therapy at one month in the stent group.

Stenting versus medical treatment in patients with symptomatic vertebral artery stenosis: a randomized open-label phase 2 trial (VAST), Compter A, et al. [15]

Compter et al. conducted an open-label, randomized, phase two clinical trial for Vertebral Artery Stenting Trial (VAST). This study was conducted at seven separate medical centers in the Netherlands from January, 2008 to April, 2013. VAST investigated the level of risk associated with PTAS plus BMT versus BMT alone in patients with symptomatic vertebral artery stenosis. Patients were managed by a vascular neurologist and an interventional neuroradiologist. The interventional neuroradiologist was required to have completed over 50 interventional procedures involving the carotid or vertebral arteries in the last five years. These requirements were established to eliminate confounding variables and ensure standardization of the competency levels in recruited physicians.

Inclusion criteria for this study included 1) patients ≥ 40 years of age, 2) TIA or non-disabling stroke of posterior cerebral circulation that occurred within the last 180 days, 3) readily available for a stenting procedure within two weeks of presentation, 4) diagnosis of vertebral artery stenosis of 50% or greater with duplex ultrasound along with CTA, MRA, or cerebral angiography, 5) presumed stenosis due to an atherosclerotic plaque, 6) calculated mRS of < 4, and 7) presence of lesion that is accessible with endovascular methods. Exclusion criteria for the study included 1) cause of TIA or stroke due to a reason other than atherosclerosis, 2) vertebral artery dissection, 3) previous endovascular surgery, 4) life expectancy of three years or less, 5) active illness, 6) severe renal impairment (complications with imaging contrast), 7) allergy to iodinated contrast, and 8) pregnancy.

Randomization of patients into the PTAS plus BMT or BMT only cohorts was completed at a 1:1 ratio and facilitated by a web-based randomization with minimization algorithm to insure limited variability amongst potential confounding variables between the two cohorts. Randomization was also stratified by medical center and location of stenosis lesion; a lesion was considered extracranial if located in the first three segments of the vertebral artery (V1-3), and considered intracranial if located in the fourth segment of the vertebral artery (V4).

All patients in the study received BMT (if deemed necessary by the attending neurologist) for risk factors that would otherwise affect the recovery and outcome of their disease, including antihypertensives, cholesterol-lowering treatment, antiplatelet treatment, and oral anticoagulants. All patients were treated daily for five days prior to surgery with anti-platelet therapy (75 mg of clopidogrel in addition to ASA) or a vitamin K antagonist. This treatment regimen was continued until 30 days after the procedure. If the patient was not on clopidogrel the day before the procedure, the patient was administered 300 mg of clopidogrel prior to the PTAS procedure. Patients in the PTAS plus BMT cohort were to undergo PTAS only if the procedure was feasible or not contraindicated. The choice of stent type was decided by the interventional radiologist performing the procedure. Follow-up evaluations were completed in person at one month and 12 months after initiation of BMT or the day of PTAS. Follow-ups were completed by telephone at six months and on a yearly basis after the 12-month in-person visit. Patients were evaluated one day after PTAS to assess the patency of the newly-stented artery and focal neurological deficits. Duplex ultrasound was completed at three months status post PTAS to assess the long-term patency of the stented artery. At 12 months, all patients underwent duplex ultrasound and CTA to assess for long-term patency of the stented artery in the PTAS cohort and progression of stenosis in patients enrolled in the BMT only cohort. Restenosis was defined as any residual or recurrent stenosis of at least 50% or occlusion of the vertebral artery on CTA during follow-up.

The enrolled patients and participating physicians were not blinded to treatment, so an independent masked endpoint committee reviewed the results for analysis of primary and secondary outcomes. If there was any suspicion to the validity of an outcome, the event was reviewed by three neurologists who were asked to provide an anonymous written evaluation of the outcome. If all three reviewed did not align, then the majority opinion was utilized as the official consensus.

The primary outcome was defined as the composite of vascular death, myocardial infarction, or any stroke within 30 days after the start of treatment. Among those who carried a diagnosis of intracranial vertebral artery stenosis and were enrolled in the PTAS plus BMT cohort, 22% of patients (2/9) experienced a cardiovascular event consistent with the primary outcome definition. In comparison, those who carried a diagnosis of intracranial vertebral artery stenosis and only received BMT, 0% (0/10) experienced a cardiovascular event consistent with the primary outcome definition. The investigators hypothesized that PTAS plus BMT may be a promising technique over BMT alone to prevent recurrent vascular events in the vertebrobasilar territory. Although the overall data including extracranial and intracranial vertebral artery stenosis supports PTAS (primary outcome of 5% in PTAS plus BMT versus 3% in BMT alone), the analysis of those with intracranial vertebral artery stenosis is not supportive of PTAS. This data aligns with the general conclusions reported by the SAMMPRIS and VISSIT clinical trials.

Critiques of the study include the low number of patients who were diagnosed with intracranial vertebral artery stenosis and therefore low power for this portion of the study. For this reason, it is difficult to make definitive conclusions on the level of risk and prognosis associated with PTAS plus BMT in comparison to BMT alone for those with symptomatic intracranial vertebral artery stenosis. The analysis resulted in similar demographics between cohorts for the majority of variables considered, except for the number of patients within 14 days of a previous cardiovascular event and with an mRS that is greater than one at inclusion. Studies have reported that the risk of complications from a TIA or stroke is highest within 14 days of the cardiovascular incident [26]. A study completed by Rantner et al. displayed a similar increased, short-term risk in a cohort of patients who underwent carotid artery stenting over those who underwent carotid endarterectomy [27]. Gulli et al. reported an increased risk of early recurrent stroke (within one month of initial event) in those with vertebrobasilar stenosis, which highlights the importance of early intervention, whether it is BMT or PTAS. This is contrary to the traditional theory that posterior circulation strokes have a low correlation with the risk of recurrent strokes [26]. The percentage of patients enrolled in the study within 14 days of the cardiovascular incident among the BMT only cohort (36%) was higher than in the PTAS plus BMT cohort (28%). Although this difference was not statistically significant, it may have slightly reduced the perceived overall risk with performing PTAS plus BMT in comparison to BMT alone. The mRS is utilized to score patients on their level of disability. The percentage of patients with an mRS > 1 among the BMT only cohort (34%) was higher than in the PTAS plus BMT cohort (16%, p < 0.05). Lastly, there is uncertainty as to the quality of management of cardiovascular risk factors within the trial as the median systolic blood pressure was 150 mmHg. Improper management of risk factors has the potential to impact the progression of the patient’s clinical picture that was otherwise thought to be under control.

Stenting versus medical treatment for severe symptomatic intracranial stenosis, Tang CW, et al. [22]

Tang et al. conducted a randomized single-center controlled trial of 114 patients in Taiwan that compared the long-term outcomes of severe ICAS treated with PTAS plus BMT to BMT alone. BMT was determined to be double antiplatelet therapy consisting of 81-325 mg ASA and 75 mg clopidogrel as well as guideline-based management of blood pressure, HbA1c, and cholesterol for the entire follow-up duration (median follow-up duration 17.6 months). Two neuroradiologists blinded to patient treatment group assignment cooperatively managed vascular events that enrollees may have experienced during the study.

The decision to enroll a patient in this study was made by a council of neuroradiologists, neurologists, and neurosurgeons. Patients were randomly assigned to one of the two treatment groups; 53 patients received PTAS in addition to BMT while six patients received BMT only. All patients enrolled had severe ICAS (70%-99% stenosis). There were no significant differences in comorbidities, demographics, and risk factors between enrolled patients or treatment groups, confirming the success of patient matching. However, the authors indicated that the prevalence of extracranial stenosis was higher in BMT only enrollees than PTAS plus BMT patients (54% versus 36% of patients, respectively). Outcomes were measured by the number and rate of adverse clinical events. Adverse clinical events were characterized by the mRS to be a consequence of minor or major intracranial artery ischemia. Minor events are associated with a lower mRS score of <4, while major events were associated with an increased mRS score of ≥4.

Tang et al. showed that at one-year and three-year follow-up, the proportion of patients that experienced an adverse clinical event was similar in both treatment groups: 23% at one year and 26.6% at three years in patients receiving PTAS plus BMT versus 22% at one year and 24.6% at three years in patients receiving BMT alone. However, the rate of vascular events in the BMT only group was higher than that of the PTAS plus BMT cohort (9.8% versus 3.2%, respectively, p = 0.31). The timeline of vascular events also differed between the two treatment groups, as the PTAS plus BMT group experienced more vascular events within the first postoperative week (75% of all treatment group vascular events) than the BMT only group (17% of all treatment group vascular events). On the other hand, the incidence of vascular events was higher after the one-year follow-up in the BMT only cohort (19.7%) than in PTAS plus BMT cohort (3.8%). At the three-year follow-up, a greater percentage of the PTAS plus BMT cohort was asymptomatic (30.2% with an mRS = 0) than the BMT only cohort (13.1% mRS = 0). The PTAS plus BMT cohort also experienced less disabling vascular events at three years (5.7% with an mRS score = 4-6) than the BMT only cohort (11.5% mRS = 4-6).

While there were no differences in the total adverse events between the two treatment groups, Tang et al. demonstrated that there were fewer major, disabling events in PTAS plus BMT than the BMT only cohort. Thus, the investigators conclude that PTAS with BMT is associated with more favorable long-term outcomes than BMT alone. However, the authors suggest that the improved outcomes associated with PTAS plus BMT may be attributed to the greater percentage of patients with extracranial stenosis in the BMT only group.

Strengths of this study include the randomized design and losing no patients to follow-up. Potential weaknesses of the study include single-site data collection and the presence of a pertinent confounding variable that was not accounted for during stratification (extracranial stenosis).

Enterprise stent for the treatment of symptomatic intracranial atherosclerotic stenosis: an initial experience of 44 patients, Feng Z, et al. [28]

As shown in the SAMMPRIS and VISSIT trials, deployment of the Wingspan and balloon-expandable stent in patients with symptomatic ICAS has a high risk of periprocedural complications, especially when stenosis is in a complex setting such as long lesions (>15 mm), tortuous vascular pathways, and/or arterial bifurcations. This study aims to assess the efficacy and safety of undersized balloon angioplasty followed by deployment of the highly flexible, self-expanding Enterprise stent in patients with complex symptomatic ICAS.

This study is a retrospective, single-center analysis of 44 patients with symptomatic ICAS that were treated with combined antiplatelet therapy, intensive risk factor management, and balloon angioplasty followed by Enterprise stent deployment. Selected patients had stenosis >70% via DSA using formulas described by the WASID method, recurrent TIAs or ischemic stroke despite BMT, and stenosis located in complex settings. Patients with total occlusive lesions, severe disability because of stroke or dementia, or inability to give informed consent were excluded.

Deployment of the Enterprise stent had success rate of 100%. Four (9.1%) major complications occurred during the periprocedural period (30 days), which included three (6.8%) ischemic perforator strokes and one (2.2%) reperfusion hemorrhagic stroke. In the 42 patients available at a median 25.6 (range, 12-57) month follow-up, there were no further TIAs or strokes. Of the 38 patients who underwent angiographic follow-up, three (6.81%) developed >50% in-stent restenosis after a mean 22-month follow-up.

This study demonstrates that undersized balloon angioplasty followed by Enterprise stent deployment is technically successful with a low rate of complications in patients with symptomatic ICAS located in complex areas. Feng et al. reported higher procedural success rates, lower periprocedural strokes, and lower in-stent re-stenosis (ISR) rates than published results for the Wingspan and balloon-expandable stents. However, this conclusion is limited by the retrospective design, small sample size, and short-term follow-up of this study, which dramatically increases the effect of selection bias.

Current literature has not adequately assessed high-risk patient subgroups such as patients that are hemodynamically unstable or have a history of multiple ischemic events while on BMT. Moreover, present efforts to reduce periprocedural complications of PTAS such as perforator artery infarction, reperfusion-related intracerebral hemorrhage, and delayed in-stent restenosis are inadequate. Elucidating the mechanisms behind these adverse events may help optimize PTAS outcomes by informing improved methods of selecting the appropriate balloons, stents, and patients. For instance, the rigidity and high radial force of the Wingspan stent used in the SAMMPRIS trial is ill-equipped for ICAS in tortuous vascular segments, long lesions, and arterial bifurcations; these complex symptomatic ICAS patients may have better outcomes with the Enterprise stent, which is more flexible and exerts less radial force. Also, ICAS patients with risk factors for ISR (e.g., long lesions or uncontrolled diabetes) may benefit from the selection of drug-eluting angioplasty and/or stents, which have been shown to reduce ISR rates relative to their bare metal counterparts.

Overall, this study did provide a proof-of-concept for future trials focused on tailoring device selection to specific types of ICAS patients in order to investigate a potential reduction in perioperative complications. The China Angioplasty and Stenting for Symptomatic Intracranial Severe Stenosis Trial is an ongoing, multicenter RCT that will attempt to address some of these issues. Prospective, multicenter RCTs with a larger sample size and longer follow-up period are needed.

## Conclusions

This review illustrates that PTAS in the setting of symptomatic ICAS is associated with worse short and long-term outcomes, including increased rates of ischemic stroke, hemorrhagic strokes (reperfusion injury), and death when compared to BMT alone. The association between PTAS and worse outcomes persisted when examined solely in patients with symptomatic stenosis of intracranial vertebral arteries. Large, multicenter RCTs examining the efficacy of PTAS plus BMT in ICAS patients with hemodynamic instability and/or symptoms refractory to BMT are needed to further define the role of PTAS. Prospective, head-to-head studies of different stent types are also required. Our findings validate the current American Heart Association and American Stroke Association recommendations that PTAS should not be employed as first-line treatment in patients with symptomatic ICAS. However, PTAS may be considered in symptomatic ICAS patients that are hemodynamically unstable or have repeatedly failed BMT therapy regimens.
